# Plastomes of two *Rehmannia* species： comparative genomic and phylogenetic analyses

**DOI:** 10.1080/23802359.2021.1878953

**Published:** 2021-03-11

**Authors:** Jing Sun, Mengqi Sun, Danchou Wang, Kailun Xu, Renyong Hu, Yonghua Zhang

**Affiliations:** a College of Life and Environmental Sciences, Wenzhou University, Wenzhou, China; b National and Local Joint Engineering Research Center of Ecological Treatment Technology for Urban Water Pollution, Wenzhou University, Wenzhou, China

**Keywords:** Chloroplast genome, phylogenomics, *Rehmannia glutinosa*, *Rehmannia chingii*, Rehmannieae

## Abstract

Phylogenetic relationships within *Rehmannia* have not been well solved. Here, we assembled and reported two new complete plastomes of *R. glutinosa* and *R. chingii* by *de novo* assembly. The complete plastomes of *R. glutinosa* and *R. chingii* were 153,797 and 153,328 bp in length, respectively. These two plastomes had 98.8% sequence identity and a total of 401 SNPs, 137 indels and 6 inversions. They were highly conserved in GC content (43.1%), gene order, and gene content (133 genes), including 88 protein-coding genes, 37 tRNA genes, and 8 rRNA genes. The phylogenomic analysis confirmed the monophyly of Rehmannieae and supported *R. chingii* as the basal taxon of *Rehmannia.*

*Rehmannia* Libosch. ex Fisch. et Mey., a small genus of tribe Rehmannieae (Orobanchaceae), is endemic to China (Rix 1987; Zeng et al. 2017). *R. glutinosa* (Gaert.) Libosch. ex Fisch. et Mey. is distributed from North China to Korea (Rix 1987), whose root is usually used as traditional medicine (Kim et al. 2008; Zhang et al. 2008). *Rehmannia chingii* H.L. Li, located in Tianmu mountains and adjacent areas of Southeast China, was seriously threatened by human activities (Zeng et al. 2016). Although the phylogenetic tree of all six *Rehmannia* species with complete plastomes has been reconstructed before, the phylogenetic relationship within *Rehmannia* remains controversial (Xia et al. 2009; Zeng et al. 2017). Here, we reported two new plastomes of *R. glutinosa* and *R. chingii* with further comparative genomic and phylogenomic analyses.

Samples of *R. glutinosa* and *R. chingii* were collected from Dahongzai (Henan, China, 34°14′16.91″N, 113° 5′23.44″E) and Mt. Tianmu (Zhejiang, China, 30°19′9.23″N, 119°26′33.20″E), respectively. Voucher specimens (*Minqi Cai CMQ16223*, *Yonghua Zhang ZYH160425*) were deposited in the Herbarium of Wenzhou University (WZU). Genomic DNAs were extracted from silica-dried leaves with modified CTAB method (Doyle and Doyle 1987). Then 125-bp Paired-end reads were sequenced on the Illumina HiSeq^2500^ platform. Complete plastomes were assembled and confirmed using NOVOPlasty (Dierckxsens et al. 2017) and CLC Genomics Workbench 11 (Zhang et al. 2019; Liu et al. 2020). Gene annotation was performed using program PGA (Qu et al. 2019) with *Rehmannia henryi* (GenBank accession numbers: KX636158) as a reference.

The complete plastomes of *R. glutinosa* and *R. chingii* (GenBank accession numbers: MW013795, MW013796) are 153,797 and 153,328 bp in genome sizes, respectively. Their plastomes contain a large single copy (LSC) region (84,672–84,257 bp), a small single copy (SSC) region (17,607–17,587 bp), and a pair of inverted repeats (IRs) (25,759–25,742 bp). The two plastomes have 98.8% sequence identity and a total of 401 SNPs, 137 indels and 6 inversions. They are highly conserved in GC content (43.1%), gene order, and gene content (133 genes, including 88 protein-coding genes, 37 tRNA genes, and 8 rRNA genes).

Maximum likelihood (ML) analysis was conducted on a dataset that included 113 genes for 14 taxa (13 Orobanchaceae species, one Lamiaceae species as the outgroup) using RAxML-HPC BlackBox (8.2.12) on CIPRES (http://www.phylo.org). The phylogeny of Orobanchaceae was well resolved and supported *R. chingii* (from Mt. Tianmu) as the basal taxon of the genus (Figure 1), which is consistent with the phylogenetic result using DNA fragments (Xia et al. 2009). Compared with the study of Zeng et al. (2016, 2017), we think that the former sequenced plastome of *R. chingii* (collected from Lishui, Zhejiang, China; GenBank accession number KX426347) was most likely misidentified based on our field investigation.

**Figure 1.**
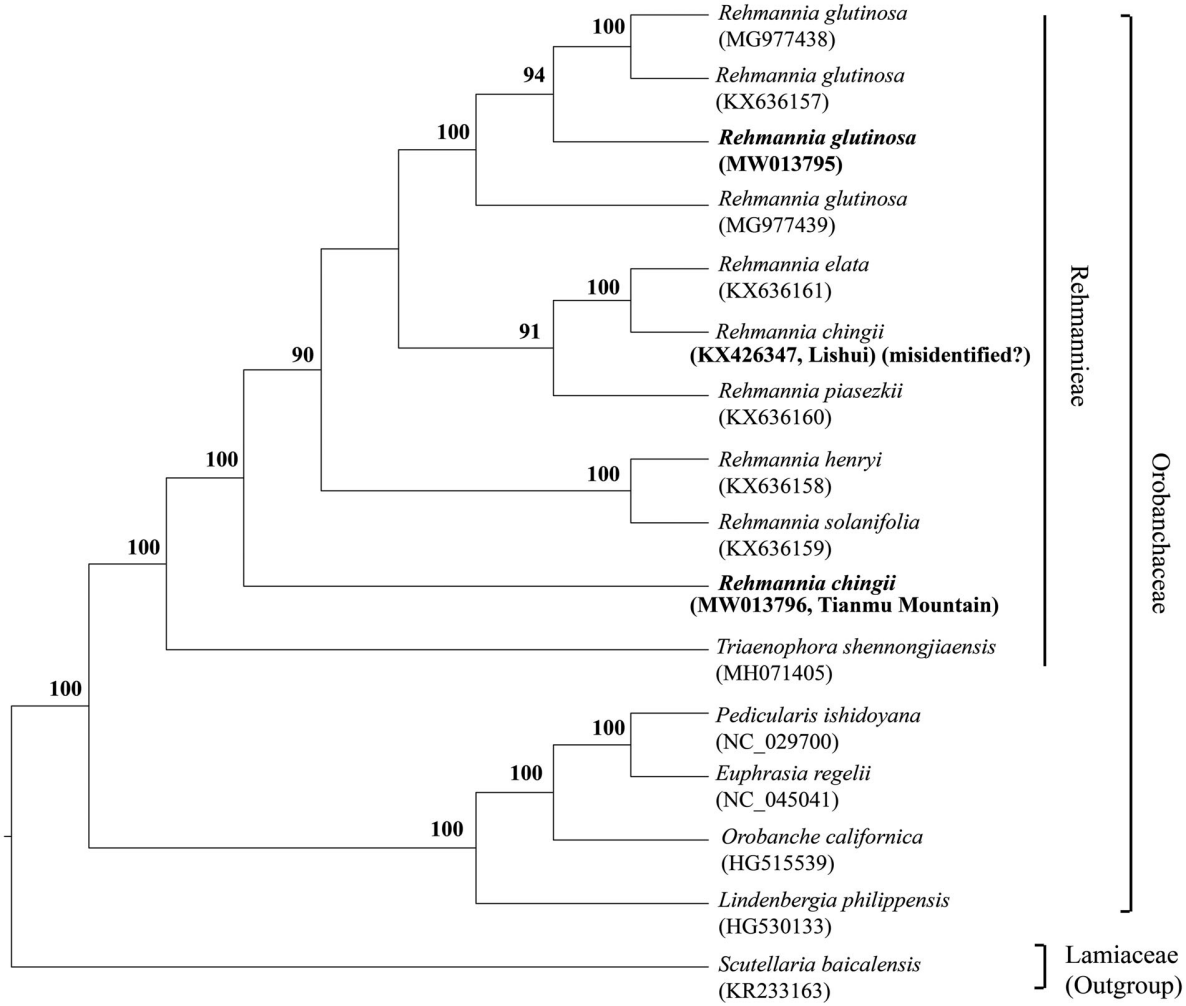
Phylogenetic tree of 14 taxa based on 113 genes of their plastomes using maximum likelihood (ML) method. Numbers near the nodes represent ML bootstrap value (≥90).

## Data Availability

The genome sequence data of *Rehmannia glutinosa* and *Rehmannia chingii* that support the findings of this study are openly available in GenBank of NCBI at (https://www.ncbi.nlm.nih.gov/) under the accession no. MW013795-MW013796. The associated BioProject and Sequence Read Archive (SRA) numbers are PRJNA664281, SRR12667759, and PRJNA664316, SRR12667977, respectively.
